# Compound Heterozygous *SCN5A* Mutations in Severe Sodium Channelopathy With Brugada Syndrome: A Case Report

**DOI:** 10.3389/fcvm.2020.00117

**Published:** 2020-07-24

**Authors:** Aleksandra Nijak, Alain J. Labro, Hans De Wilde, Wendy Dewals, Steve Peigneur, Jan Tytgat, Dirk Snyders, Ewa Sieliwonczyk, Eline Simons, Emeline Van Craenenbroeck, Dorien Schepers, Lut Van Laer, Johan Saenen, Bart Loeys, Maaike Alaerts

**Affiliations:** ^1^Center of Medical Genetics, Antwerp University Hospital, University of Antwerp, Antwerp, Belgium; ^2^Laboratory of Molecular, Cellular and Network Excitability, Department of Biomedical Sciences, University of Antwerp, Antwerp, Belgium; ^3^Department of Paediatric Cardiology, Antwerp University Hospital, Antwerp, Belgium; ^4^Department of Invasive Cardiology and Electrophysiology, Ghent University Hospital, Ghent, Belgium; ^5^Toxicology and Pharmacology, University of Leuven (KU Leuven), Leuven, Belgium; ^6^Department of Cardiology, Antwerp University Hospital, University of Antwerp, Antwerp, Belgium; ^7^Department of Human Genetics, Radboud University Medical Centre, Nijmegen, Netherlands

**Keywords:** Brugada syndrome, cardiac arrhythmia, *SCN5A*, case report, compound heterozygosity

## Abstract

**Aims:** Brugada syndrome (BrS) is an inherited cardiac arrhythmia with an increased risk for sudden cardiac death (SCD). About 20% of BrS cases are explained by mutations in the *SCN5A* gene, encoding the main cardiac sodium Na_v_1.5 channel. Here we present a severe case of cardiac sodium channelopathy with BrS caused by *SCN5A* compound heterozygous mutations. We performed a genetic analysis of *SCN5A* in a male proband who collapsed during cycling at the age of 2 years. Because of atrial standstill, he received a pacemaker, and at the age of 3 years, he experienced a collapse anew with left-sided brain stroke. A later ECG taken during a fever unmasked a characteristic BrS type-1 pattern. The functional effect of the detected genetic variants was investigated.

**Methods and Results:** Next-generation sequencing allowed the detection of two *SCN5A* variants in *trans*: c.4813+3_4813+6dupGGGT—a Belgian founder mutation—and c.4711 T>C, p.Phe1571Leu. A familial segregation analysis showed the presence of the founder mutation in the proband's affected father and paternal aunt and the *de novo* occurrence of the p.Phe1571Leu. The functional effect of the founder mutation was previously described as a loss-of-function. We performed a functional analysis of the p.Phe571Leu variant in HEK293 cells alone or co-expressed with the β_1_-subunit. Compared to the *SCN5A* wild type, p.Phe1571Leu displayed a hyperpolarizing shift in the voltage dependence of inactivation (loss-of-function), while the activation parameters were unaffected. Using the peptide toxin nemertide α-1, the variant's loss-of-function effect could be restored due to a toxin-dependent reduction of channel inactivation.

**Conclusion:** This is the first report providing support for the pathogenicity of the p.Phe1571Leu *SCN5A* variant which, together with the c.4813+3_4813+6dupGGGT founder mutation, explains the severity of the phenotype of cardiac sodium channelopathy with BrS in the presented case.

## Introduction

Brugada syndrome (BrS) is an inherited cardiac arrhythmia with a significant risk for sudden cardiac death (SCD) and a prevalence of 1:2,000 in the general population (1). The disease is diagnosed based on a specific ECG pattern with distinct ST-segment elevation in the right precordial leads (2, 3). The disorder is predominantly explained by mutations in *SCN5A* (20–25% of all cases), encoding the α-subunit of the cardiac voltage-gated sodium channel Na_v_1.5. Currently, over 20 genes encoding other cardiac channels as well as their accessory proteins are linked to BrS (2, 4, 5).

Cardiac action potential generation and conduction velocity in the ventricles rely primarily on the availability of Na_v_1.5 channels (3, 6), responsible for a fast depolarization of the cardiomyocyte membrane (7, 8). Na_v_1.5 is a pseudo-tetramer consisting of four repetitive transmembrane domains (DI–IV), containing six transmembrane-spanning segments each (S1–S6), with segments S1–S4 forming the ‘voltage sensing domain (VSD)’. The VSDs of DI to DIII control the channel opening and closure (activation process), whereas the VSD of DIV regulates the channel inactivation (3, 9, 10). An accessory β_1_-subunit binds covalently to the α-subunit, with variable effects on the activation kinetics (11). BrS is associated with a loss-of-function of Na_v_1.5, resulting in reduced sodium current (*I*_Na_), impaired channel kinetics, or trafficking (3–5). Several reports show that heterozygous *SCN5A* variants cause a BrS phenotype with variable expressivity, ranging from asymptomatic to recurrent arrhythmias and SCD. In addition, they can lead to cardiac conduction disease, sick sinus syndrome, dilated cardiomyopathy, and familial atrial fibrillation or an “overlap syndrome” of these entities, together grouped as cardiac sodium channelopathies. Interestingly, second-hit mutations in genes encoding the auxiliary subunits of Na_v_1.5 or compound heterozygous mutations in *SCN5A* tend to cause more severe phenotypes (4–6).

Here we present a patient with a severe phenotype of cardiac sodium channelopathy with BrS phenotype presenting in early childhood due to compound heterozygous *SCN5A* mutations. One of the variants, c.4813+3_4813+6dupGGGT—a Belgian founder mutation causing cardiac conduction defects and/or BrS in 83% of carriers (Sieliwonczyk et al., under review), segregates in the paternal family. This splice site mutation results in the deletion of 32 amino acids (1,572 to 1,604) in S2 and S3 of DIV of Na_v_1.5 with a loss-of-function of the channel, which is observed as the absence of sodium current when expressed in human embryonal kidney TSA cells (12, 13). We hypothesize that the second *de novo* variant, c.4711T>C (p.Phe1571Leu), located in S2 of DIV, aggravates the phenotype in the proband. In this study, we functionally characterize this p.Phe1571Leu variant. We provide evidence for a loss-of-function effect which, in co-occurrence with the Belgian founder mutation, most likely explains the severity of the observed phenotype.

## Materials and Methods

### Mutation Analysis of *SCN5A*

Genomic DNA was extracted from whole blood using standard procedures. Genetic testing of *SCN5A* was performed using an in-house developed cardiac arrhythmia gene panel (14). Sanger sequencing was used to validate the variants and perform familial segregation analysis. This study was carried out in accordance with the recommendations of the Ethics Committee of Antwerp University Hospital. All subjects gave written informed consent in accordance with the Declaration of Helsinki.

### Site-Directed Mutagenesis and Transfection of HEK293 Cells

WT human *SCN5A* cDNA was cloned into the pSP64T plasmid and human *SCN1B* (the β_1_-subunit) cDNA into a pRcCMV plasmid. The p.Phe1571Leu variant was introduced in *SCN5A* using the QuickChange Site-Directed Mutagenesis Kit (Life Technologies) and a primer set that contained the variant (Eurogentec S.A.). The variant *SCN5A* p.Phe1571Leu plasmid was obtained by amplification in XL2 blue cells (Agilent Technologies) and subsequently purified (purification kit, Macherey-Nagel). The presence of the desired and absence of additional mutation(s) was confirmed by sequencing.

HEK293 cells were transiently transfected with WT *SCN5A* (expressing Na_v_1.5) or the p.Phe1571Leu variant (Na_v_1.5-F1571L) alone or co-expressed in a 1:1 mass ratio with *SCN1B* using Lipofectamine 2000 (Life Technologies). In every condition, the pEGFP1-N1 plasmid was co-transfected to visualize the transfected cells for electrophysiological analysis. The cells were grown in Dulbecco's Modified Eagle Medium supplemented with 10% fetal bovine serum and 1% penicillin/streptomycin (Life Technologies). The cells were placed in a 5% CO_2_ incubator at 37°C for 48 h prior to the patch-clamp recordings.

### Electrophysiological Recordings

Whole-cell patch-clamp recordings were performed at room temperature (20–22°C) using an Axopatch 200B amplifier and a pClamp 10.7/Digidata 1440A acquisition system (Axon Molecular Devices). Patch-pipettes with a resistance between 1 and 1.5 MΩ were pulled from 1.2-mm borosilicate glass capillaries (World Precision Instruments, Inc.) using a P-2000 puller (Sutter Instrument Co.). The pipettes were filled with an intracellular solution containing (in mM): 4 NaCl, 106 KCl, 5 K_2_ATP, 2 MgCl_2_, 5 K_4_BAPTA, and 10 HEPES adjusted to pH 7.2 with KOH. The cells were continuously superfused with a bath solution (ECS) containing (in mM): 145 NaCl, 4 KCl, 5.3 CaCl_2_, 1 MgCl_2_, 10 HEPES, and 10 glucose (pH 7.35 with NaOH). For the toxin experiments, the nemertide 1-α synthetic peptide was directly dissolved in ECS at a concentration of 5 μM and applied near the patched cell using a pressurized fast-perfusion system.

*I*_Na_ was recorded by step depolarization for 20 ms to different potentials between −120 and +40 mV, from a holding potential of −130 mV. Current–voltage relations (in pA/pF) were obtained by normalizing the peak *I*_Na_ amplitude to the cell capacitance and plotting these values as a function of the applied potential. Normalized conductance–voltage (*G*–*V*) curves were obtained by approximating the linear part of the current–voltage relation with the function $I=G_{\max}^{*}\left(V_{applied}-V_{reversal}\right)$ to determine the maximal conductance *G*_max_. Dividing the data points of the current–voltage relation by the calculated maximum current at that voltage, using *G*_max_, yielded the *G*–*V* relation. Activation and inactivation kinetics were determined by fitting the activating part or decay of *I*_Na_ with a single exponential function. The voltage dependence of inactivation was determined by stepping after a 500-ms pre-pulse, ranging from −135 to −30 mV, to a −10-mV test pulse to determine the amount of channel inactivation. The voltage dependence of the inactivation curves were obtained by plotting the normalized *I*_Na_ amplitude upon the test pulse against the corresponding pre-pulse potential. The recovery from inactivation was investigated upon a 500-ms conditioning pre-pulse to induce inactivation. The recovery was determined by stepping after the pre-pulse for variable time to −130 mV, a potential that recovers inactivation, followed by −10 mV pulse to evaluate the *I*_Na_ amplitude, which is a measure for the channels that have recovered. The speed of recovery from inactivation was evaluated by normalizing the *I*_Na_ elicited upon the −10 mV pulse to the maximum *I*_Na_. These normalized values were plotted as a function of the duration at −130 mV, and this relation was approximated with a single exponential function.

Currents, after passing a 5-kHz low-pass filter, were digitized at a sampling rate of 10 kHz. Recordings were discarded from analysis if the remaining voltage error, originating from series resistance error, exceeded 5 mV after compensation.

### Data Analysis

To obtain the midpoint potential (*V*_1/2_) and the slope factor (*V*_s_) for the voltage dependence of activation and of inactivation, the *G*–*V* and voltage dependence of the inactivation curves were fitted with a Boltzmann equation: $f\left(V\right)=\frac{I_{\max}}{1+e^{-\left({V-V}_{1/2}\right)/V_{s}}}$.

Data are reported as mean ± standard error of mean (SEM) with *n* as the number of cells analyzed. A comparison between the WT and the variant was performed with two-tailed Student's *t*-test. A *P*-value of ≤0.05 indicates statistical significance (Systat Software Inc.).

## Case Description and Diagnostic Assessment

### Clinical Presentation of Index Patient and Available Family Members

**Figure 1:**
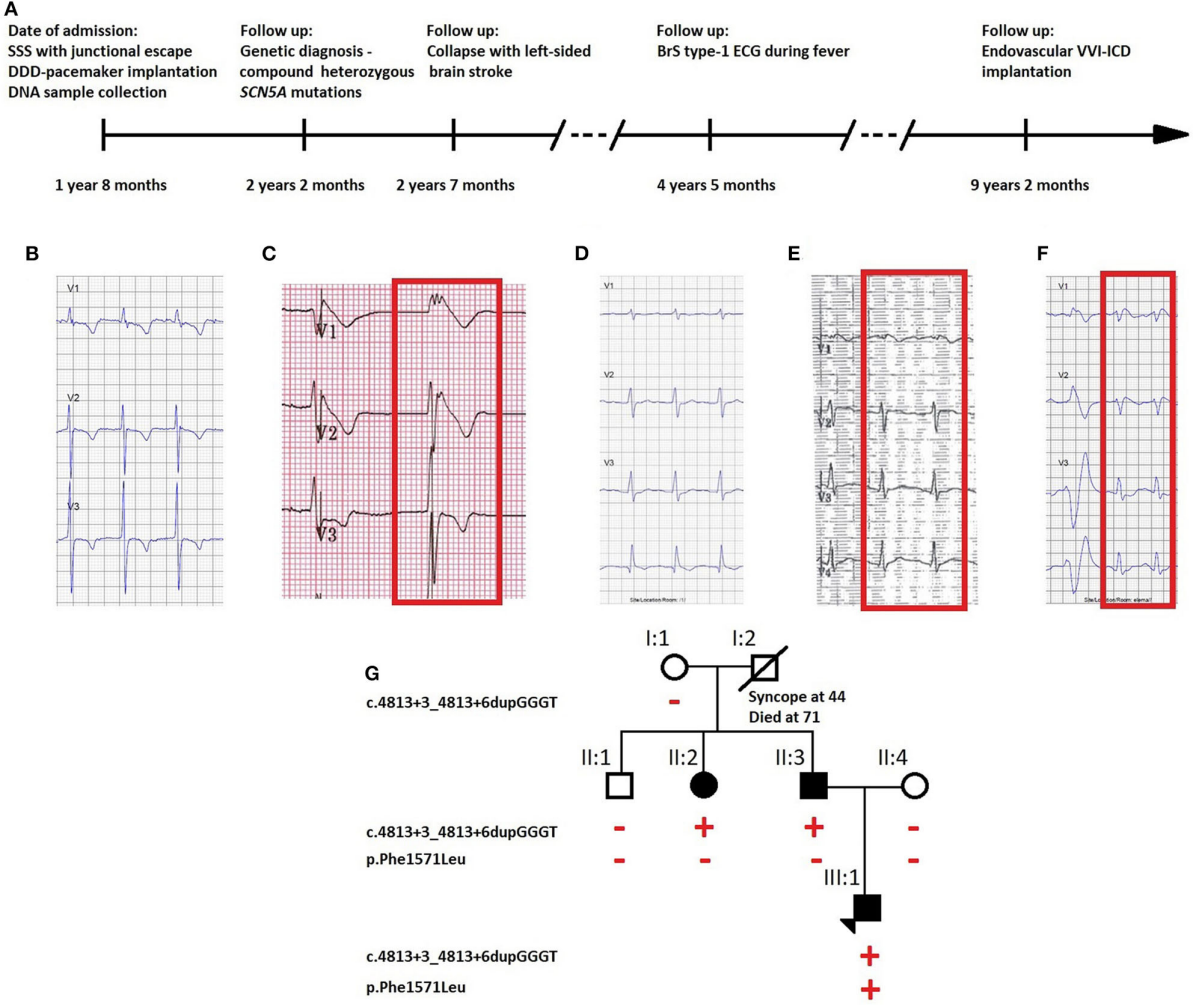
**(A)** Timeline of diagnostic interventions for the described case. ECG traces of **(B)** the proband on the day of diagnosis with absent atrial activity with junctional escape, **(C)** repeated during fever at 39°C which unmasked BrS ECG pattern with elevated ST segment, **(D)** the father carrying the *SCN5A* founder mutation with absent BrS ECG type-1 pattern after ajmaline challenge, but **(E)** present during fever at 39°C (visible in lead V1), and **(F)** the asymptomatic aunt carrying the*SCN5A* founder mutation with ECG type-1 pattern after ajmaline challenge. **(G)** Pedigree of the family showing the segregation of the identified variants. Full symbols indicate the affected individuals. The red plus and minus symbols indicate, respectively, the presence or the absence of the variant indicated on the left side of the pedigree.

A 20-months-old boy was admitted to a hospital emergency service after his collapse during cycling. Based on the ECG, he was diagnosed with sick sinus syndrome (SSS) with junctional escape (Figure 1B). A DDD-pacemaker was implanted, and during the implantation, the dilated atria were electrically inactive, leading to the diagnosis of atrial standstill. A DNA sample was collected for molecular diagnostics.

During recovery, he experienced a collapse anew, caused by a thrombus which led to a left-sided brain stroke. The echocardiography showed left atrial spontaneous echocardiographic contrast but no obvious thrombi. Although during follow-up no ventricular tachyarrhythmias were documented, an ECG taken during fever (39°C) unmasked the characteristic BrS type-1 pattern (Figure 1C), and the boy received an endovascular VVI-ICD as there is a high estimated risk of developing ventricular arrhythmias (timeline: Figure 1A).

The father had a history of syncope at the age of 30. His ajmaline challenge test was negative, but an ECG taken during a fever episode showed a BrS coved-shape ECG pattern (Figures 1D,E). The sister of the father was diagnosed with an ajmaline-induced BrS type-1 ECG pattern (Figure 1F).

### Molecular Genetics

Two *SCN5A* variants were identified in the index patient: c.4813+3_4813+6dupGGGT, a Belgian founder mutation, and c.4711T>C p. (Phe1571Leu). Since both *SCN5A* variants are separated by only 102 base pairs, we could derive from the sequence reads that they are never located on the same allele (*in trans*). No other variants of interest were detected with the PED MASTR Plus gene panel. A familial segregation analysis revealed the founder mutation in the proband's father (II:3) and paternal aunt (II:2) and *de novo* occurrence of the p.Phe1571Leu variant in the index patient (III:1) (Figure 1G).

**Figure 2:**
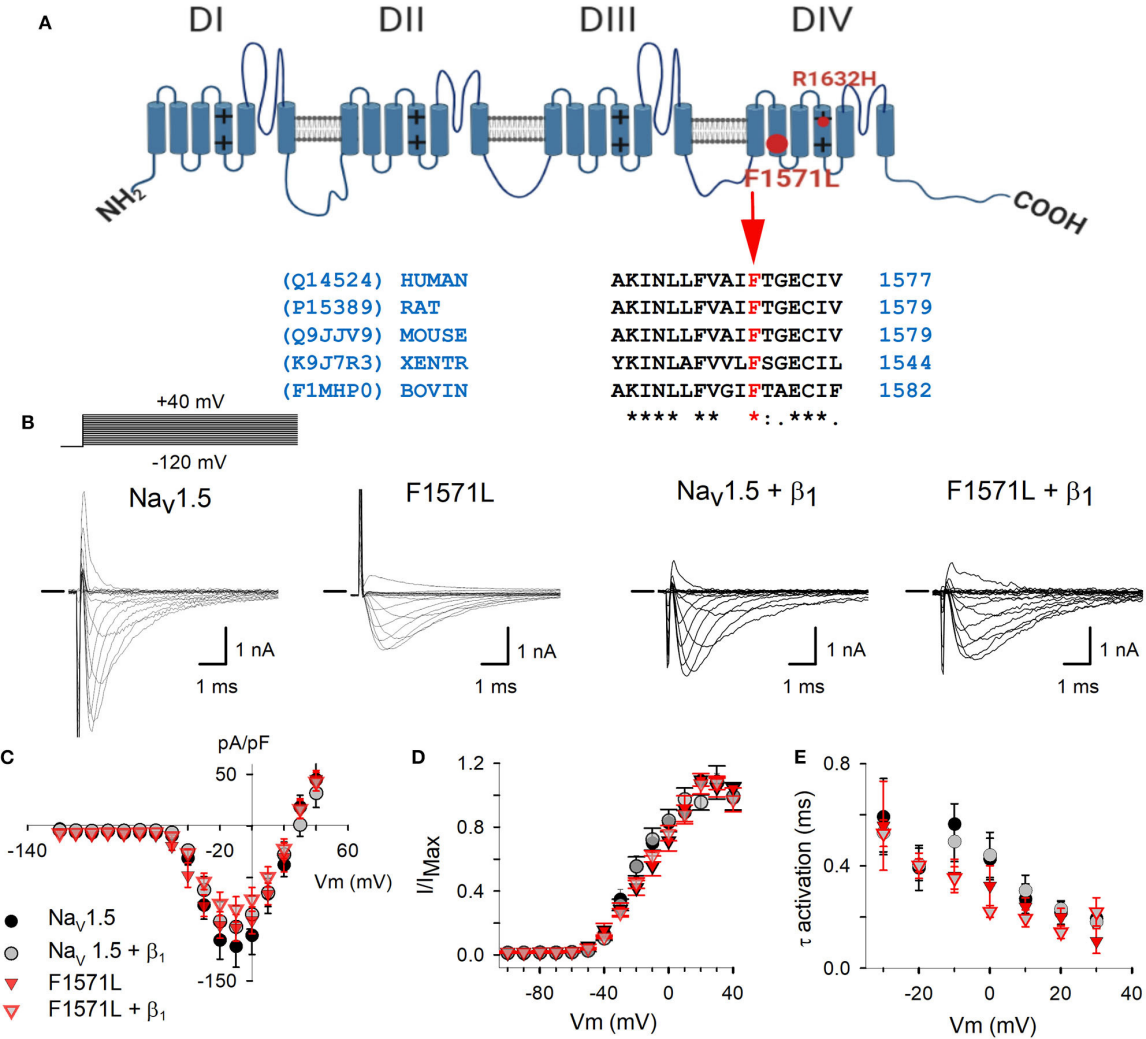
**(A)** Schematic representation of the Na_v_1.5 structure (10) with location of the p.Phe1571Leu variant (big full red circle) and p.Arg1632His (small red circle). The alignment shows the conservation of Phe at position 1571 in *SCN5A* amino acid sequence between species. The accession numbers for the UniProt database are indicated on the left side. **(B)** Electrophysiological properties of WT Na_v_1.5 and Na_v_1.5-F1571L alone or co-expressed with β_1_. Displayed from left to right are the representative whole-cell ionic current recordings of WT Na_v_1.5, Na_v_1.5-F1571L, WT Na_v_1.5 + β_1_, and Na_v_1.5-F1571L + β_1_. The Na^+^-selective currents were elicited with the pulse protocol shown on the left above the traces. The horizontal bar at the beginning of the traces indicates zero current level. **(C)** Current density of WT Na_v_1.5 (black circles, *n* = 8), WT Na_v_1.5 + β_1_ (gray circles, *n* = 9), Na_v_1.5-F1571L (full red inverted triangles, *n* = 7), and Na_v_1.5-F1571L + β_1_ (open red inverted triangles, *n* = 9) were obtained by normalizing the peak current amplitudes from pulse protocols shown in **(B)** to the cell capacitance. **(D)** Voltage dependence of channel activation, *G*–*V* curves, are represented for WT Na_v_1.5, Na_v_1.5-F1571L, WT Na_v_1.5 + β_1_, and Na_v_1.5-F1571L + β_1_. **(E)** The voltage-dependent kinetics of channel activation are shown as means ± SEM.

The founder mutation was previously shown to cause a loss-of-function of the Na_v_1.5 channel (13) and is classified as a pathogenic variant (15). p.Phe1571Leu is absent from the GnomAD database [gnomad.broadinstitute.org (16)], whereas one carrier is reported in the TOPMED sample collection (dbSNP rs1369632373; nhlbiwgs.org). Phenylalanine at position 1,571 is highly conserved between species (Figure 2A), and *in silico* prediction on the functional impact of the variant is possibly damaging (MutationTaster, SIFT, PolyPhen; ENST00000413689.5). This initially led to the classification of this variant as a variant of uncertain significance (VUS) (15).

### Electrophysiological Characteristics

To determine if this VUS contributes to the severe phenotype observed in our index patient, the Na_v_1.5 WT and Na_v_1.5-F1571L variant and combinations with the WT β_1_-subunit (Na_v_1.5 + β_1_ and Na_v_1.5-F1571L + β_1_) were expressed in HEK293 cells to assess the functional effect of the variant. The current–voltage (*I*–*V*) relationship showed that the current densities for the Na_v_1.5 and Na_v_1.5-F1571L, either expressed alone or in presence of β_1_, did not differ (Figures 2B,C). The activation kinetics as well as the voltage dependence of channel activation (*G*–*V* curves) did not show significant differences, yielding a *V*_1/2_ of −13.8 ± 1.7 and −14.9 ± 0.9 mV, with slope factors of 14.5 ± 2.4 and 14 ± 1.3 mV for Na_v_1.5 and Na_v_1.5-F1571L, respectively (Figures 2D,E). These values did not change significantly in the presence of β_1_, yielding *G*–*V* curves with a *V*_1/2_ of −12 ± 0.8 and −14.7 ± 1.1 mV, with slope factors of 16.1 ± 1 and 11.7 ± 1.7 mV for Na_v_1.5 + β_1_ and Na_v_1.5-F1571L + β_1_, respectively.

**Figure 3:**
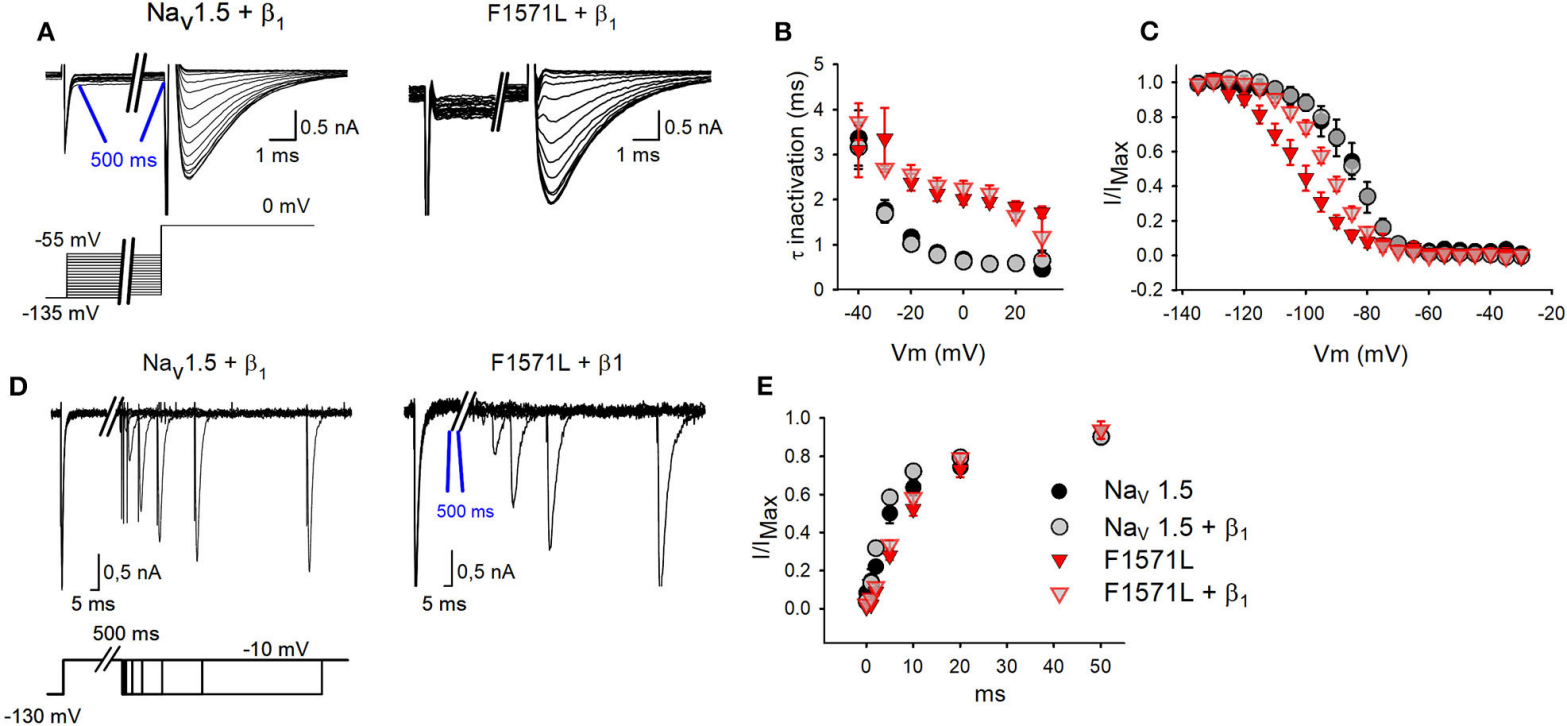
Inactivation properties of WT Na_v_1.5 and Na_v_1.5-F1571L alone or co-expressed with β_1_. **(A)** Displayed from left to right are the representative whole-cell inactivation recordings of WT Na_v_1.5 + β_1_ and Na_v_1.5-F1571L + β_1_. The *I*_Na_ were elicited with the pulse protocol shown underneath the traces. **(B)** The time constants of channel inactivation for WT Na_v_1.5 (black circles, *n* = 5), WT Na_v_1.5 + β_1_ (gray circles, *n* = 12), Na_v_1.5-F1571L (full red inverted triangles, *n* = 9), and Na_v_1.5-F1571L + β_1_ (open red inverted triangles, *n* = 12). The values shown are means ± SEM. **(C)** Voltage dependence of channel inactivation obtained by plotting the normalized current amplitudes at −10 mV, elicited after 500 ms of conditioning pre-pulse depolarization, as a function of the pre-pulse potential. **(D)** Displayed from left to right are the representative whole-cell recordings of recovery from inactivation of WT Na_v_1.5 + β_1_ and Na_v_1.5-F1571L + β_1_. The protocol used is shown underneath the traces. **(E)** Graph representing the recovery from inactivation, sampled after 500 ms from induction of inactivation.

We subsequently analyzed the kinetics of inactivation, the voltage dependence of inactivation, and the recovery from inactivation (Figures 3A–E). From the inactivation time constant, we observed a 3-fold slower inactivation (*P* < 0.001) for the variant (1.96 ± 0.12 ms at 0 mV, *n* = 7) compared to Na_v_1.5 WT (0.67 ± 0.08 ms at 0 mV, *n* = 8) (Figure 3B). Co-expression with β_1_ did not compensate this slowing, and Na_v_1.5-F1571L + β_1_ (2.25 ± 0.16 ms at 0 mV, *n* = 9) displayed 3.6-fold slower inactivation kinetics compared to WT (0.63 ±0.02 ms at 0 mV, *n* = 9) (*P* < 0.001).

Interestingly, the Na_v_1.5-F1571L variant displayed a significant 18-mV hyperpolarizing shift in the voltage dependence of inactivation compared to WT (*P* = 0.001), yielding inactivation curves with a *V*_1/2_ of −104 ± 3 mV (*n* = 9) and −86 ± 3 mV (*n* = 5), combined with a slope factor of 7.6 ±0.8 and 5.4 ±0.7 mV for Na_v_1.5-F1571L and Na_v_1.5, respectively (Figure 3C). Co-expression with the β_1_ subunit did not rescue the effect of the variant, and Na_v_1.5-F1571L + β_1_ displayed a significant 8.3-mV hyperpolarizing shift compared to Na_v_1.5 + β_1_ (*P* = 0.003), yielding inactivation curves with a *V*_1/2_ of −93 ± 2 mV (*n* = 12) and −84 ± 2 mV (*n* = 12), combined with a slope factor of 7.2 ± 0.5 and 5.8 ± 0.5 mV, respectively (*P* = 0.04).

As the onset of inactivation was slowed in the variant, we observed also a slowed recovery from inactivation. The time constants of recovery from inactivation revealed a slower recovery for the variant channel compared to WT (*P* < 0.001), which was not rescued by co-expression with the β_1_ subunit (*P* < 0.001) [Na_v_1.5: 7.4 ± 0.1 ms (*n* = 6); Na_v_1.5-F1571L: 15.2 ± 0.1 ms (*n* = 12); Na_v_1.5+ β_1_: 5.2 ± 0. 2 ms (*n* = 10); Na_v_1.5-F1571L + β_1_: 13 ± 0.1 ms (*n* = 15)] (Figures 3D,E).

**Figure 4:**
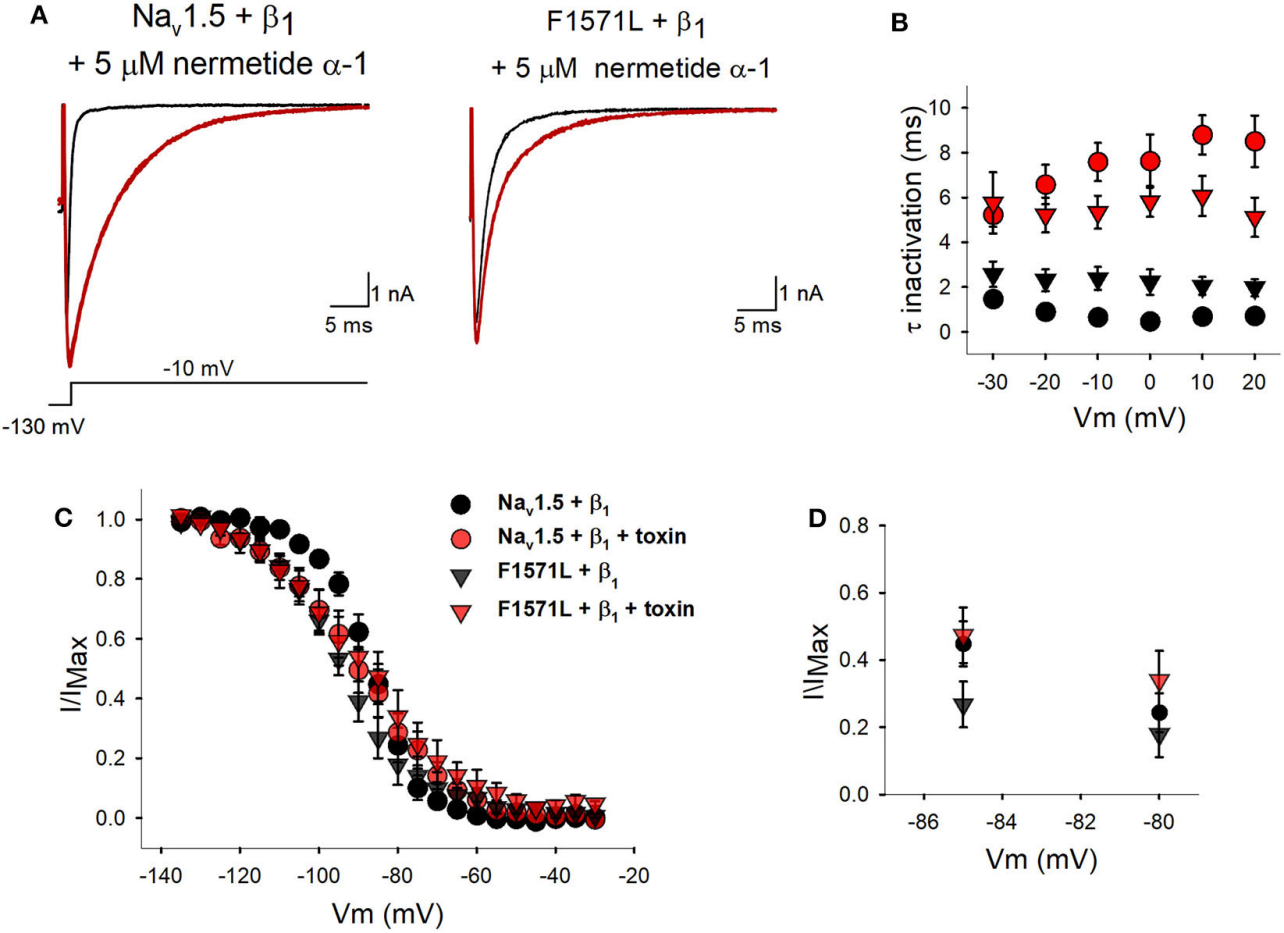
Inactivation properties of WT Na_v_1.5 + β_1_ and Na_v_1.5-F1571L +β_1_ in the presence of nemertide α-1. **(A)** Displayed from left to right are the representative whole-cell recordings of WT Na_v_1.5 + β_1_ and Na_v_1.5-F1571L + β_1_ in control conditions (black tracings) and upon steady-state modification by 5 μM nemertide α-1 (red tracings). The currents were elicited with the pulse protocol shown underneath the traces. Steady-state toxin modulation was achieved after ~2 min. **(B)** Kinetics of channel inactivation for WT Na_v_1.5 + β_1_ (circles) and Na_v_1.5-F1571L + β_1_ (inverted triangles) in control conditions (black) and upon steady-state modification by nemertide α-1 (red) are shown. The values are means ± SEM. **(C)** The voltage dependence of channel inactivation for WT Na_v_1.5 + β_1_ (black symbols) and Na_v_1.5-F1571L + β_1_ (red) in control conditions (circles) and upon steady-state modification by nemertide α-1 (inverted triangles) is shown. **(D)** Scaled-up view of the voltage dependence of channel inactivation in the physiological range of resting membrane potentials, with a focus on the comparison to the WT Na_v_1.5 + β_1_. Note that, in the presence of nemertide α-1, the amount of inactivated Na_v_1.5-F1571L + β_1_ channels (red inverted triangles) is similar to the WT in control conditions (black circles).

As this patient's severe phenotype seems to be associated with Na_v_1.5-F1571L displaying a hyperpolarizing shift in the voltage dependence of inactivation, we explored if we could restore the variant's voltage dependence of inactivation toward WT values by drug/toxin addition. The peptide toxin nemertide α-1 seemed to be a good candidate as it was reported to have a decelerating effect on the inactivation process of human sodium channels without influencing the parameters of activation and *I*_Na_ density (17). Two minutes of perfusion of 5 μM of nemertide α-1 induced a steady-state modification of *I*_Na_ inactivation of both Na_v_1.5 + β_1_ and the variant p.F1571L + β_1_ (Figure 4A). Upon this nemertide α-1 addition, we observed a 16-fold slowing in the inactivation kinetics of Na_v_1.5+ β_1_; at 0 mV, the time constant of inactivation slowed from 0.47 ± 0.04 ms in the absence of nemertide α-1 to 7.62 ± 1.18 ms with nemertide α-1 (*n* = 6; *P* = 0.0002). The variant Na_v_1.5-F1571L + β_1_ also reported a significant 3-fold slowing of inactivation (*P* = 0.002) displaying at 0 mV a time constant of 2.22 ± 0.57 ms in the absence of nemertide α-1 and 5.82 ± 0.68 ms in its presence (*n* = 6) (Figure 4B). Next, we investigated the voltage dependence of inactivation and observed no significant changes in the *V*_1/2_ upon toxin addition for both Na_v_1.5+β_1_ and Na_v_1.5-F1571L+ β_1_ (*P* > 0.5). In contrast, the slope factors became significantly shallower and amounted, after toxin modification, to 10.6 ± 0.8 mV (*n* = 6) and 12.3 ± 1.3 mV (*n* = 6) for Na_v_1.5 + β_1_ and Na_v_1.5-F1571L + β_1_, respectively (Figures 4C,D). Due to the shallower slope, the amount of Na_v_1.5-F1571L + β_1_ channel inactivation is reduced at the physiological relevant resting membrane potential (i.e., around −85 mV) upon nemertide α-1 addition.

## Discussion

BrS is rarely observed in the pediatric population (18–20), and only 4.3% of symptomatic patients experience their first malignant ventricular arrhythmic event before the age of 16 years (21). Single heterozygous loss-of-function variants in *SCN5A* can cause BrS, cardiac conduction disease, sick sinus syndrome, dilated cardiomyopathy, and familial atrial fibrillation or manifest as an ‘overlap syndrome’ of these entities (22). When *SCN5A* is affected by two compound variants, this can result in a significant aggravation of the disease severity and/or earlier disease onset (5, 23–26). This was also observed in our proband who experienced his first syncope during physical activity at around the age of 2 years, probably caused by brady-arrhythmia in the setting of atrial standstill. Most of the similarly published patients presented with sinus node dysfunction (5, 23, 24, 26). Only Sacilotto et al. reported a patient presenting initially with atrial flutter and recurrent syncopes without spontaneous ventricular arrhythmia and who, similarly to our case, presented a spontaneous BrS type-1 ECG pattern during follow-up (24). Clinical interventions mostly encompass the implantation of a pacemaker (5, 23, 24, 26), with additional low-dose aspirin (24) or oral quinidine (23), and single patient received β-blockers without pacemaker implantation (25).

In the presented family, heterozygous carriers of the *SCN5A* Belgian founder mutation showed characteristic BrS type-1 ECG pattern either after sodium channel blocker challenge (II:2) or during fever conditions (II:3) but presented no symptoms (II:2) or syncope at the age of 30 years (II:3). The proband's second variant, p.Phe1571Leu, is a missense variant that we functionally modeled in HEK293 cells. Our experiments showed that p.Phe1571Leu does not affect current density, voltage dependence of channel activation, or activation kinetics (Figures 2C–E). However, it did impair the inactivation properties, as could be expected from the localization of the variant in the VSD of DIV, which is known to regulate channel inactivation (9). On the one hand, the variant slows down inactivation (Figure 3B), which would suggest a gain-of-function of the variant. This effect would rather be predicted to lead to a long-QT syndrome type 3 phenotype (4) but, on the other hand, the voltage dependence of inactivation of p.Phe1571Leu displays a significant hyperpolarizing shift (Figure 3C) which, in physiological conditions, results in a reduction of over 40% in the availability of Na_v_1.5 channels at rest (i.e., at −85 mV). The significantly slowed recovery of the mutant channel (Figure 3E) further reduces the availability of the remaining channels, exacerbating the effect. This combination obviously leads to a loss-of-function of *I*_Na_, explaining the (aggravated) phenotype of cardiac sodium channelopathy with BrS in the patient.

Interestingly, an almost identical electrophysiological effect was reported for the *SCN5A* p.Arg1632His (R1632H) variant (5, 26), located in DIV S4 (Figure 2A) in proximity to p.Phe1571Leu. Slower inactivation kinetics, delayed recovery from inactivation, and a hyperpolarizing shift in voltage dependence of inactivation of similar magnitude (20.7 mV) as we observed (18 mV) were described (26). The heterozygous carriers of this variant had no symptoms, SSS, ajmalin-induced BrS (5), or first-degree AV block (25), while the compound heterozygous carriers (with second *SCN5A* variant) presented with early-onset SSS (5, 25). This supports the likely pathogenicity of the p.Phe1571Leu variant characterized in this study and its causal role in aggravating the phenotype of the presented case.

Also interesting is that another missense variant at the same amino acid location, p.Phe1571Cys, has been reported in a Brugada syndrome patient (27), but no functional analysis was performed. Although the resulting amino acid change is different, this could be supportive of our findings as well.

Our experiments with the toxin nemertide α-1 showed that the WT channels were responding as expected as inactivation kinetics were slowed down. However, no depolarizing shift in the voltage dependence of inactivation was observed for the WT Na_v_1.5+β_1_ as was observed for Na_v_1.5 expressed in *Xenopus laevis* oocytes (16). This might be because of a different cell type and/or the addition of the β_1_-subunit, which seems to protect the cells from fluctuations in the voltage dependence of inactivation. Nevertheless, toxin addition resulted in a shallower voltage dependence of inactivation (i.e., larger slope factor value) such that the amount of inactivated Na_v_1.5-F1571L channels is reduced at the physiological resting membrane potential (Figures 4C,D). In the presence of toxin, the amount of available non-inactivated Na_v_1.5-F1571L + β_1_ channels rises from 18% without toxin to ~36% (at −85 mV), which is similar to the amount of available WT Na_v_1.5 + β_1_ channels. To our knowledge, this is the first report in which the use of a sodium channel activator toxin is proposed as a potential remedy for the pathogenic effect of a *SCN5A* variant.

Based on these functional experiments, we conclude that the described p.Phe1571Leu variant is likely pathogenic (15) and, in the presented case, its *de novo* occurrence, together with the *SCN5A* Belgian founder mutation, explains the severe phenotype of cardiac sodium channelopathy with BrS.

## Data Availability Statement

The datasets presented in this study can be found in online repositories. The names of the repository/repositories and accession number(s) can be found below: https://www.ncbi.nlm.nih.gov/, SCV001190331 https://www.ncbi.nlm.nih.gov/, SCV001190333.

## Ethics Statement

The studies involving human participants were reviewed and approved by Ethics Committee of the Antwerp University Hospital. Written informed consent to participate in this study was provided by the participants' legal guardian/next of kin. Written informed consent was obtained from the individual(s), and minor(s)' legal guardian/next of kin, for the publication of any potentially identifiable images or data included in this article.

## Author Contributions

AN, AL, BL, and MA designed and planned the experimental framework. AN performed the experiments. AN, AL, and MA wrote the manuscript with critical input from all the authors. AN analyzed the data under the supervision of AL. JT and SP advised and provided the toxin for the functional experiments. HD, WD, EV, LV, and JS provided the clinical data of the index patient and family members.

## Conflict of Interest

The authors declare that the research was conducted in the absence of any commercial or financial relationships that could be construed as a potential conflict of interest.
